# Discharge prescription patterns of opioid and nonopioid analgesics after common surgical procedures

**DOI:** 10.1097/PR9.0000000000000637

**Published:** 2018-02-06

**Authors:** Michael J. Nooromid, Eddie Blay, Jane L. Holl, Karl Y. Bilimoria, Julie K. Johnson, Mark K. Eskandari, Jonah J. Stulberg

**Affiliations:** a Division of Vascular Surgery, Feinberg School of Medicine, Northwestern University, Chicago, IL, USA; b Department of Surgery, Feinberg School of Medicine, Northwestern University, Chicago, IL, USA; c Center for Healthcare Studies, Institute for Public Health and Medicine, Feinberg School of Medicine, Northwestern University, Chicago, IL, USA

**Keywords:** Opioid, Postoperative pain, Surgery, Discharge prescription

## Abstract

**Introduction:**

Recent literature has shown a wide variation in the prescribing patterns of opioids after elective surgery. We conducted an evaluation of discharge opioid prescribing after elective surgical procedures to determine whether opioid-prescribing patterns varied at our institution.

**Method:**

A single academic medical center retrospective review of patients undergoing laparoscopic cholecystectomy, laparoscopic appendectomy, open umbilical hernia repair, simple mastectomy, or thyroidectomy between July 2015 and July 2016.

**Results:**

Among a total of 615 unique surgical cases, we found a wide variation in the number of pills and morphine milligram equivalents prescribed for each procedure evaluated. In addition, 94.8% of all patients discharged received a prescription for opioids, whereas only 15.6% of patients received a prescription for a nonopioid analgesic.

**Conclusion:**

The number and strength of opioids prescribed after surgery can vary widely at a single institution. Further research is needed to elucidate variations in prescribing.

## Abstract 2

The number and strength of opioids prescribed after surgery can vary widely at a single institution. Further research is needed to elucidate variations in prescribing.

## 1. Introduction

Many surgeons prescribe opioids to manage postoperative pain, which was highlighted in a recent study evaluating American College of Surgeons National Surgical Quality Improvement Program data that found 93.9% of the 7651 included surgical patients had received an opioid prescription at discharge.^11^ Meanwhile, it has been shown that the quantity of opioids prescribed after selected low-risk surgical procedures has increased with time.^12^ The trends characterized in postoperative opioid prescribing are concerning because the risk of opioid naïve patients to develop persistent opioid use, defined as opioid use 90 to 180 days after surgery, was found to be 5.9% after minor surgery and 6.4% after major surgery in a population-based study of 36,177 patients.^2^

Despite the increasing societal burden of opioid abuse, there is currently no evidence-based guidelines for the treatment of postoperative pain with opioids. This has been shown to lead to a significant variation in the number of pills and quantity of opioids prescribed at discharge.^3,11^ This article characterizes variation in postoperative opioid and nonopioid analgesic prescribing for common surgical procedures at a large academic medical center.

## 2. Methods

We conducted a retrospective cohort study using the Northwestern Medicine Enterprise Data Warehouse of patients discharged for the 5 most commonly performed operations at our hospital: laparoscopic appendectomy, laparoscopic cholecystectomy, open umbilical hernia repair, simple mastectomy, and thyroidectomy. All opioid, benzodiazepine, and nonopioid analgesic medications (ie, nonsteroidal anti-inflammatory drugs, acetaminophen, or gabapentin) prescribed at discharge were quantified and categorized. For opioids, the number of tablets and total morphine milligram equivalents (MMEs) prescribed for each discharge prescription were calculated. Institutional Review Board approval was obtained for this study.

## 3. Results

Six hundred fifteen distinct discharges were identified between July 1, 2015, and July 31, 2016. The mean age of all included patients was 47.3 years (range, 18–82). Three hundred eighty-eight (63.1%) patients were women and 227 (36.9%) were men. In total, we found that 94.3% of all patients discharged received a prescription for opioids, whereas only 15.6% of patients received a nonopioid prescription (Table 1). Opioids prescribed included hydromorphone, hydrocodone, oxycodone, codeine, and tramadol, whereas nonopioid analgesics prescribed included acetaminophen, gabapentin, ibuprofen, and naproxen.

**Table 1:**
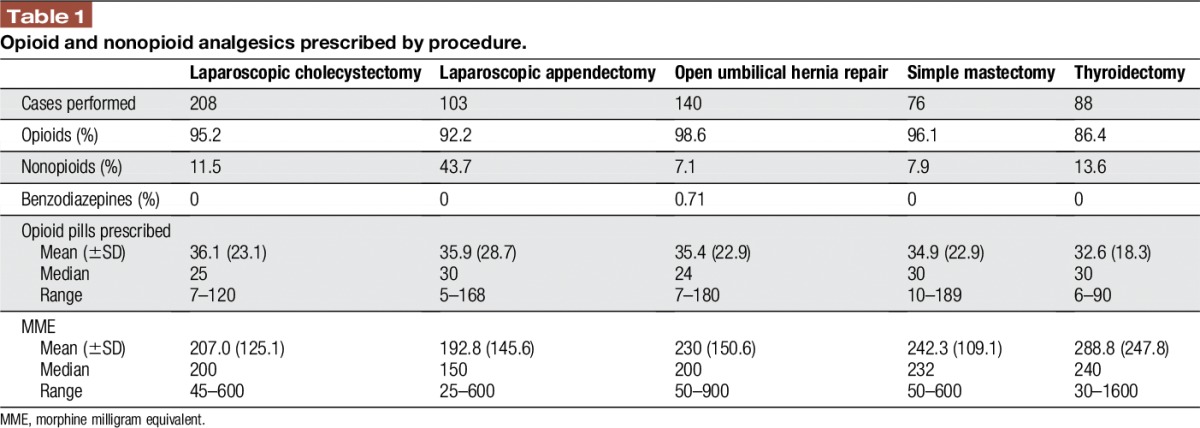
Opioid and nonopioid analgesics prescribed by procedure.

Overall, the highest proportion of patients receiving an opioid prescription (98.6%) and the lowest proportion receiving a nonopioid prescription (7.1%) occurred after open umbilical hernia repair. The median number of opioid pills prescribed was between 24 for open umbilical hernia repair and 30 for laparoscopic appendectomy, simple mastectomy, and thyroidectomy. The median MME prescribed after these operations ranged from 150 to 240 MME, with the smallest dose of opioids prescribed after a laparoscopic appendectomy. Amongst all prescriptions, the range of MME varied 64-fold, from 25 to 1600 MMEs.

## 4. Discussion

Previous work has shown a variation of opioid prescribing by geographic location, race, and individual centers.^6–8^ A recent study by Hill et al.^3^ found a wide variation in discharge opioid prescribing among 5 common general surgical procedures and also noted that only 28% of the pills prescribed were actually taken by patients. Similarly, the overprescribing of opioid analgesics has also been characterized in multiple surgical specialties including orthopedic surgery, urology, and oral surgery.^1,4,5^ The results of our study confirm significant variation in opioid prescribing across several common general surgery procedures with only minimal use of nonopioid medication, at our institution.

Although a Canadian study found that the 1-year risk of opioid use after major surgery in opioid naïve patients was only 0.4%, this problem is amplified on a national scale, given that there were 17.2 million ambulatory hospital visits or inpatient stays in 2014 in the United States that included an invasive therapeutic surgery.^9,10^ Currently, no surgical society has endorsed best practice guidelines for opioid and nonopioid analgesic prescribing after surgery, and this has created an environment where the number of tablets and total MME prescribed to patients is variable by provider. To diminish the variability in opioid prescribing and encourage alternative pain therapy, guidelines, education, and postoperative prescribing guidelines and education are critically needed.

Our study has notable limitations associated with a retrospective review. First, we were not able to determine which patients had previous opioid use or account for each patient's pain tolerance. Likewise, the discharge prescriptions were not adjusted for surgical complexity, postoperative complications, medical comorbidities, length of hospital stay, type of anesthesia, or distance traveled from the hospital, which could have had significant influence.

## 5. Conclusion

There is a wide variation in the number of pills and MMEs of opioids prescribed after general surgery procedures. In addition, there are significantly lower percentages of nonopioids prescribed as part of multimodal pain therapy at discharge. Further work is needed to determine how the use of multimodal pain therapy at discharge effectively manages postoperative pain and develops best practice guidelines for postsurgical pain therapy.

## Disclosures

The authors have no conflict of interest to declare.

M. J. Nooromid and E. Blay are partially supported by a National Institutes of Health Grant (T32HL094293-06). The content is solely the responsibility of the authors and does not necessarily represent the official views of the National Institutes of Health. J. J. Stulberg is the primary investigator of a grant (R34DA044752) from the National Institute on Drug Abuse of the National Institutes of Health titled “System-Level Implementation to Reduce Excess Opioid Prescribing in Surgery.” The content is solely the responsibility of the authors and does not necessarily represent the official views of the National Institutes of Health. J. J. Stulberg is the primary investigator of a grant from the Digestive Health Foundation (www.digestivehealthfoundation.org) titled “A Multidisciplinary Collaboration to Minimize Diversion of Opioids.” The content is solely the responsibility of the authors and does not necessarily represent the official views of the Digestive Health Foundation.
